# Counteractive Effects of Copper Nanoparticles and Betacellulin on Ovarian Cells

**DOI:** 10.3390/nano14231965

**Published:** 2024-12-07

**Authors:** Alexander V. Sirotkin, Paula Romero-Navarro, Barbora Loncová, Zuzana Fabová, Michaela Bartušová, Abdel Halim Harrath, Francisco Alonso

**Affiliations:** 1 Department of Zoology and Anthropology, Constantine the Philosopher University, Tr. A Hlinku 1, 949 74 Nitra, Slovakia; barboraloncova@gmail.com (B.L.); zuzka.fabova@gmail.com (Z.F.); zfabova@ukf.sk (M.B.); 2 Instituto de Síntesis Orgánica (ISO) and Departamento de Química Orgánica, Facultad de Ciencias, Universidad de Alicante, Apdo. 99, 03080 Alicante, Spain; paula.romero@ua.es; 3 Department of Zoology, College of Science, King Saud University, Riyadh 11451, Saudi Arabia; hharrath@ksu.edu.sa

**Keywords:** apoptosis, betacellulin, copper, nanoparticles, ovary, proliferation

## Abstract

Copper nanoparticles (CuNPs) are known to affect many ovarian cell functions. CuNPs, prepared using a chemical reduction method, were fully characterized by different means (TEM, DLS, XRD, Z potential, XPS, and AES). The resulting colloidal suspension contained needle-like CuNPs aggregates made of a core of metallic copper and an oxidized surface of Cu_2_O and CuO. The separate and coupled effects of CuNPs and the growth factor betacellulin (BTC) were analyzed on the control of some basic functions of ovarian cells. With this purpose, porcine ovarian granulosa cells, together with CuNPs, BTC, and both (CuNPs + BTC), were cultured. Viability and BrDU tests, quantitative immunocytochemistry, TUNEL, and ELISA were used to evaluate markers of the S-phase (PCNA) and G-phase (cyclin B1) of the cell cycle, cell proliferation (BrDU incorporation), cytoplasmic/mitochondrial apoptosis (bax) and extrinsic (nuclear DNA fragmentation) markers, and the release of estradiol and progesterone. CuNPs were accumulated within the cells and were found to reduce all the markers of proliferation, but promoted all the markers of apoptosis and the release of steroid hormones. When added alone, BTC raised the expression of all cell viability and proliferation markers, depleted the expression of all apoptosis markers, and stimulated the release of both estradiol and progesterone. Furthermore, BTC prevented and even reversed the effect of CuNPs on all the measured parameters, whereas CuNPs mitigated BTC’s effect on all the analyzed cell functions. These results support a direct toxic effect of CuNPs and a stimulatory effect of BTC on ovarian cell functions, as well as the capability of BTC to protect against the adverse effects of CuNPs.

## 1. Introduction

Copper nanoparticles (CuNPs) are widely used in scientific techniques, medicine, and animal production [1,2,3]. It is well known that some CuNPs can promote ovarian cell functions [4,5,6,7]. Most of the reported CuNPs have been proven to be environmental contaminants with toxic effects on non-ovarian [2,8,9] and ovarian [4,5,6,10,11,12] cells. Therefore, the intentional and unintentional exposure of humans and animals to the adverse influence of harmful CuNPs cannot been ignored. Among the CuNPs studied on ovarian cells, spherical CuNPs colloids were found to be the most harmful ones. When these CuNPs were tested on cultured porcine ovarian cells, inhibition of the cell cycle, the promotion of apoptosis, and alteration of the release of some steroid hormones were observed [5].

The search for some natural/physiological protective agents of the female reproductive system against the toxic influence of CuNPs is of interest from scientific and practical points of view. Growth factors—naturally occurring substances, the primary physiological function of which is to promote cell growth, viability, and development—might have such a protective role by counteracting CuNPs and lessening their reproductive toxicity. While CuNPs on follicular granulosa cells of pigs can boost the release of insulin-like growth factor I [4], the interplay between CuNPs and other growth factors, including betacellulin, in regulating ovarian cell functions has not been reported yet. Betacellulin (BTC) is a growth factor that can promote basic functions. It is known that BTC stimulates the maturation of the oocyte–cumulus complex [13,14,15], prostaglandin production [14,16], ovulation [17] and proliferation in cultured healthy [14,18] and cancer [19] ovarian cells. In contrast, BTC depleted the activity of cultured feline ovarian cells [20]. Recently, it has been reported that BTC can promote the stimulatory effect of some CuNPs on ovarian cells [7]; the nanoparticles were immobilized on a charcoal support. However, as far as we know, the ability of BTC to protect ovarian cells from the adverse effects of CuNPs has not been studied yet.

By virtue of the interest of some of our research team in new methods to obtain and stabilize metal nanoparticles [21], we prepared CuNPs colloids, which were used as chemical catalysts in different organic reactions [22,23]. The adverse impact of these CuNPs on ovarian cell functions has been shown in only one report [5], which should be validated with further studies, whereas the capability of BTC to affect porcine ovary cells and to diminish or suppress the negative influence of CuNPs on these cells has not been examined yet. Therefore, the following objectives were proposed for the present study: (1) to corroborate the inhibitory effect of CuNPs on the roles of porcine ovary cells in pigs, as formerly proven [5]; (2) to examine the effect of BTC on these cells; and (3) to study the ability of BTC to modify and to mitigate/prevent the noxious effects of CuNPs. To achieve these goals, markers of the secretory activity, apoptosis, and proliferation of porcine ovary cells were analyzed, after being cultured together with of BTC and CuNPs, either independently or together (CuNPs + BTC). A series of regulators and markers of the cell functions were analyzed, including a viability test, BrDU incorporation (cell proliferation [24]), PCNA (the mitosis S-phase [25]), cyclin B1 (the mitosis M and G-phases [24,26]), the fragmentation of DNA (nuclear DNA fragmentation, extrinsic apoptosis [27]), caspase 3 and bax (mitochondrial/cytoplasmic apoptosis [28]), and estradiol and progesterone (apoptosis, ovarian cell proliferation, ovarian folliculogenesis, oogenesis, and luteogenesis [29,30].

## 2. Materials and Methods

### 2.1. Instrumentation

Transmission electron microscopy (TEM) micrographs were obtained with a JEOLJEM2010 microscope (JEOL Ltd., Tokyo, Japan), fitted with a filament of LaB_6_ and run at 200 kV acceleration voltage. The samples were observed after being deposited on gold grids coated with a Lacey carbon film. The nanoparticle size was measured using this technique. The simultaneous measurement of Dynamic Light Scattering (DLS) and the Zeta potential was performed by combining a Nanotrac Flex (Microtrac, Haan, Germany) and Stabino II apparatus (Merkel Technologies Ltd., Yehud, Israel), from Microtrac (Verder Scientific Group) and Colloid Metrix, respectively. The Powder X-Ray Diffraction (XRD) spectrum was recorded with a Bruker D8 Advance X-ray diffractometer (Bruker, Billerica, MA, USA), in the 2*θ* mode and with Cu Kα_1_ irradiation (*λ* = 1.5406 Å), at 22 °C and 2*θ* = 2.5–80. The spectra from X-Ray Photoelectron Spectroscopy (XPS) and Auger Electron Spectroscopy (AES) were recorded with a VG-Microtech Multilab 3000 electron spectrometer (SPECSGROUP, Berlin, Germany). The instrument was fitted with a non-monochromatized Al-Kα radiation source with a power output of 300 W, and with a hemispheric electron analyzer of 9 channeltron electron multipliers. The intensities of the contributions were determined from the integral of each peak, which was calculated after removing the S-shaped baseline. The recorded curves were fitted through the association of both the Gaussian (70%) and Lorentz (30%) lines. The C 1*s* line at 284.4 eV was taken as a reference of all the binding energies, yielding values with ±0.2 eV precision.

### 2.2. Preparation of Copper Nanoparticle Suspension

Lithium powder (Medalchemy S.L., Alicante, Spain), CuCl_2_ (97%, Merck, Rahway, NJ, USA), and 4,4′-di-*tert*-butylbiphenyl (DTBB, Merck) of commercial grade were purchased. Dry tetrahydrofuran (THF) was obtained by purifying commercial THF (Merck, ACS reagent, ≥99.0%) through an alumina column in a PS-400-3MD system (Scharlab S.L., Barcelona, Spain). This was carried out using the typical procedure: A suspension made of lithium powder (14 mg, 2.0 mmol) and the electron carrier DTBB (27 mg, 0.1 mmol) in dry tetrahydrofuran (20 mL) was prepared under an argon atmosphere at room temperature. The addition of CuCl_2_ (134 mg, 1.0 mmol) to that suspension led to a mixture which changed from dark green to black, being indicative of the generation of a CuNP suspension [22]. Deionized water (10 mL) was added to a 1.9 mL aliquot from this suspension to increase the dilution.

### 2.3. Isolation and Culture of Granulosa Cells

The ovaries in this study were sourced from Landrace prepubertal gilts aged 6–8 months at a local slaughterhouse in Rastislavice (Nové Zámky, Slovakia), and were transported to the lab within 6 h of slaughter inside a thermos containing a sterile physiological 0.9% NaCl solution. Extraction of the target cells was accomplished through aspiration with a syringe from 2.5–6 mm diameter porcine ovarian follicles, with no visible signs of atresia. Following a centrifugation process (10 min, 1500 rpm), the cells were washed in a DMEM/F12 1:1 sterile medium (Dulbecco’s Modified Eagle Medium: Nutrient Mixture F12, BioWhittaker^TM^, Lonza, Basel, Switzerland), and suspended again in this medium, but we added 1% an antimycotic and antibiotic solution (Merck) and 10% bovine-fetal serum (Bio-West, Bradenton, FL, USA). The initial cell concentrations prior to the culture setup were in the range of 10^5^–10^6^ cells/mL. The cell suspension was then distributed as follows: (a) 96-well plates for bromodeoxyuridine (BrdU), terminal deoxynucleotidyl transferase dUTP nick-end labeling (TUNEL) (Brand GmbH, Wertheim, Germany), and AlamarBlue (Thermo Fisher Scientific, Waltham, MA, USA, 100 μL/well) assays; (b) 24-well plates (Nunc A/S, 1 mL suspension/well) for the enzyme-linked immunosorbent assay (ELISA) test; and (c) 16-well chamber slides (Nunc Inc., Waltham, MA, USA; 200 μL/well) for immunocytochemistry. The preculture of all the cells was carried out at 37 °C under a humidified-air atmosphere of 5% CO_2_ for two days, until a confluent monolayer of 70–80% was formed. To fix a monolayer of cells on 16-well chamber slides, 4% paraformaldehyde in PBS was used for 10 min, followed by storage at +4 °C until immunocytochemistry analysis. Following washing and fixation, the cells were incubated in the blocking solution (1% goat serum in phosphate-buffered saline, PBS) at room temperature for 1 h, to block the nonspecific binding of antiserum. Afterwards, the cells were incubated in the presence of monoclonal antibodies against either PCNA (marker of proliferation) or bax (marker of apoptosis) (all from Santa Cruz Biotechnology, Inc., Dallas, TX, USA, dilution of 1:500 in PBS) for 2 h at room temperature, and overnight at +4 °C. The aforesaid medium (humidified-air atmosphere of 5% CO_2_) was replenished with another fresh one of equal composition. Additionally, betacellulin (BTC; Sigma-Aldrich, St. Louis, MO, USA) was added (0, 100 ng/mL) to the experimental groups in a fresh medium, in the presence or absence of CuNPs (0, 1, 10, 100 ng/mL). The latter doses were in line with those utilized in earlier assays involving BTC [13,14,15,16,18,19] and CuNPs [4,5,6,8,9,10,11,12], as well as in the order of those found in the environment (e.g., natural waters, soil, etc.) [31,32,33,34]. The suspension of CuNPs and dissolution of BTC were performed in the culture medium just preceding the experiment. The control groups consisted of cells that did not receive any external treatment. Following a 48 h culture period, the cells were prepared for the TUNEL and BrdU assays and quantitative immunocytochemistry, while the medium was prepared for the ELISA (enzyme immunoassay). An automated cell counter (Thermo Fisher Scientific, Waltham, MA, USA) was used to ascertain the concentration of the cells.

### 2.4. Assessment of CuNP Accumulation by Cells

After 2 days of culture, the presence of CuNPs within the cells was inspected under the light of a microscope at a magnification of ×400. The percentage of cells containing CuNPs was counted.

### 2.5. Cell Viability Assay

The cell viability was analyzed using a Cell Counting Kit-8 (CCK-8; Abcam, Cambridge, UK) as recommended by the manufacturer. In brief, 10 µL of CCK-8 solution was added to each well, and the plates were incubated at 37 °C for 24 h. The absorbance (abs) was read at 450 nm by using an ELISA reader (Thermo Fisher Scientific, Inc., Waltham, MA, USA). The percentage of proliferative active cells was calculated.

### 2.6. BrdU Assay

The proliferation of the cells was determined by measuring the incorporation of BrdU (5-bromo-2′-deoxyuridine) during the synthesis of DNA, and was assessed by colorimetric cell proliferation ELISA (Roche Diagnostics GmbH, Mannheim, Germany), following the guidelines provided by the manufacturer. Quantitative analysis of the products was performed by using an ELISA reader (Thermo Fisher Scientific, Waltham, MA, USA) and determining the absorbance at *λ* = 450 nm.

### 2.7. TUNEL Assay

The fragmentation of DNA produced in the culture of the cells was quantified with a TUNEL assay (HT TiterTACS™ Apoptosis Detection Kit), in accordance with the guidelines provided by the manufacturer. An ELISA reader was used to record the absorbance at *λ* = 450 nm after the addition of 0.2 N HCl. The cells were marked without terminal deoxynucleotidyl transferase (TdT) for the negative control, whereas TACS-Nuclease was used for the positive controls at 37 °C for 1 h prior to treatment with hydrogen peroxide.

### 2.8. Immunocytochemical Analysis of the Presence of Proliferation and Apoptosis Markers

The markers of cell proliferation (cyclin B1 and PCNA) and apoptosis (bax) were identified using immunocytochemistry. A reported method [5,6] was followed involving primary mouse monoclonal antibodies against cyclin B1, PCNA, bax, or caspase 3 (1:500 dilution in PBS), and a secondary swine antibody against mouse IgG (1:1000 dilution), which was horseradish peroxidase-labeled (Servac). The dye 3.3′-diaminobenzidine (DAB) (Roche Diagnostics GmbH) was used to stain the cells subjected to labeling with horseradish peroxidase. The untreated cells with the primary antibody served as the negative controls. The count of stained cells was established on the basis of the brownish color of DAB peroxidase, observed under the light of a microscope. The proportion of the stained cells relative to the overall cell count was then calculated.

### 2.9. Enzyme-Linked Immunosorbent Assay (ELISA)

The progesterone and 17β-estradiol concentrations of the incubation media were measured using ELISA (25 aliquots), following the guidelines provided by the manufacturer (LDN Immunoassays and Services). The cross-reactivities of the antiserum against progesterone observed with other steroids were as follows: ≤1.1% with 11-desoxycorticosterone; ≤0.35% with pregnenolone; ≤0.30% with 17α-hydroxyprogesterone; ≤0.20% with corticosterone; ˂0.10% with estriol, 17β-estradiol, testosterone, cortisone, and 11-desoxycortisol; and ˂0.02% with DHEA-S and cortisol. The assay had a sensitivity of 0.045 ng/mL, with the coefficients of variation of the inter- and intra-assay being ˂5.59 and ˂5.40, respectively. The antiserum used against the steroid estradiol in the estradiol ELISA showed cross-reactivity of ≤9.5% with fulvestrant, ≤4.2% with estrone, ≤3.8% with E2-3-glucuronide, ≤3.6% with E2-3-sulfate, ≤0.4% with estriol, and ˂0.1% with 17-hydroxyprogesterone, androstenedione, corticosterone, E2-17-glucuronide, pregnenolone, testosterone, and progesterone. This ELISA had a sensitivity of 6.2 pg/mL, with the coefficients of variation of the inter- and intra-assay being ˂4.5 and ˂6.4, respectively. All the hormone concentration experiments were conducted in duplicate in the medium of incubation. The precision of all ELISAs was validated for cultured-medium samples through tests of dilution.

### 2.10. Statistical Analysis

The data of this study are presented as the means derived from three distinct experiments, carried out on different days using separate granulosa cell groups; six ovaries, at least, were included in each experiment. Each experimental group was characterized by four culture wells filled with gilt ovarian cells. For the immunocytochemical analyses, the ratio of cells containing the antigen was determined for a minimum of 1000 cells per well. At least 500 cells per group were inspected to evaluate nanoparticle accumulation. The optical density of the signals was quantified as a percentage relative to the control, based on the BrdU and TUNEL analyses, following the kit manufacturer’s guidelines. For the ELISA, subtraction of the blank control values from the corresponding values of the media-containing wells allowed us to eliminate any nonspecific interference, which accounted for less than 10% of the total measured values. The rates of hormone secretion were calculated per 10^6^ viable cells/day. Any significant group difference was identified with the use of one-way ANOVA and, afterwards, by Tukey’s test (SigmaPlot 11.0, Systat Software, GmbH). The differences were deemed statistically relevant at *p*< 0.05.

## 3. Results

### 3.1. Preparation and Characterization of CuNPs

**Figure 1:**
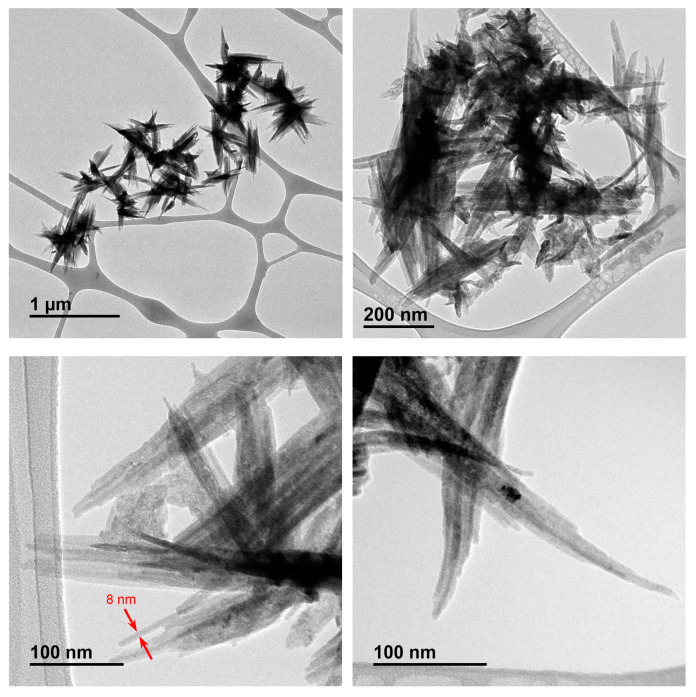
TEM micrographs of CuNPs.

**Figure 2:**
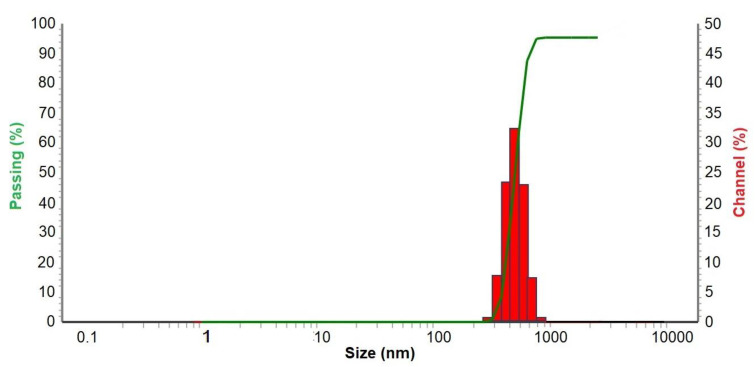
DLS graphic of CuNPs.

**Figure 3:**
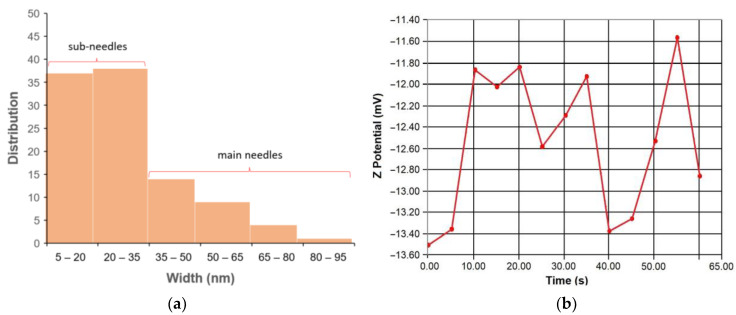
(**a**) The needle width estimated by TEM for CuNPs; the comparative distribution of main needles and sub-needles is not significant, as some of the sub-needles were measured inside the main needles. (**b**) The Z potential graphic of CuNPs.

The CuNP suspension was obtained following a previously developed methodology [21], based on the chemical reduction of CuCl_2_ with metal lithium powder and DTBB, which acts as an electron catalytic carrier [22], followed by dilution with deionized water. The as-prepared aqueous CuNP suspension was characterized by transmission electron microscopy (TEM), showing the presence of needle-like aggregates (Figure 1). A closer observation of the main needles displayed a parallel arrangement of smaller sub-needles. The size of the aggregates was determined by Dynamic Light Scattering (DLS), with 98.5% of them being in the range of 350–700 nm (Figure 2); the other parameters determined by DLS analysis were as follows: MI (Mean Intensity Diameter) = 590 nm, MA (Mean Area Diameter) = 455 nm, MN (Mean Number Diameter) = 408 nm, and median diameter = 450 nm. The width of the needles was roughly established by TEM, with the main ones being in the range of 35–95 nm and the sub-needles in the range of 5–35 nm (Figure 3a). These CuNPs differ in size and shape from those described in previous reports before dilution in deionized water (ca. 3 nm, spherical) [22] or after immobilization on a support (ca. 6 nm, spherical) [7].

The measurement of the zeta potential was used to assess the stability of our colloidal system [35]. Generally, suspensions are considered stable when the zeta potentials are above +30 mV or below –30 mV. The average zeta potential obtained for our CuNP suspension was –12 mV (Figure 3b), which pointed to a somewhat unstable system, prone to particle aggregation, something that was in agreement with the presence of the agglomerates observed by TEM.

**Figure 4:**
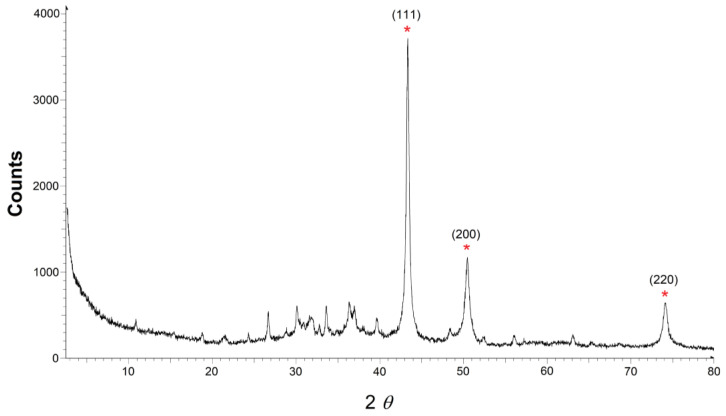
XRD spectrum of CuNPs (* denotes fcc Cu).

The XRD spectrum shows peak positions which are consistent with the main presence of face-centered-cubic copper (JCPDS file no. 04-0836) (Figure 4). Although some minor peaks might be attributable to copper oxides (Cu_2_O and CuO) [36], their presence was better confirmed at the surface of the particles on the basis of XPS analyses (see below).

**Figure 5:**
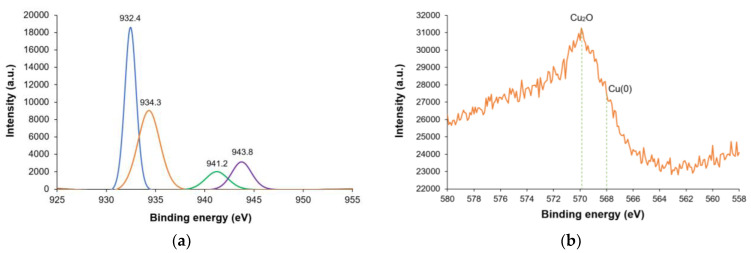
(**a**) XPS spectrum (Cu 2*p*_3/2_ level) and (**b**) CuL_3_M_45_M_45_ Auger energy spectrum of CuNPs.

The XPS spectrum of CuNPs displays Cu 2p_3/2_ peaks at 932.4, 934.3, 941.2, and 943.8 eV (Figure 5a). The peak at 934.3 eV is indicative of CuO, together with the satellite shakeup features at 941.2 and 943.8 eV. The assignment of the peak at 932.4 eV is, however, more challenging because the binding energies of Cu(0) and Cu(I) are very close (ca. 932.0–933.0 eV) [37,38]. The CuL_3_M_45_M_45_ peaks in the Auger energy spectrum were analyzed in order to distinguish between Cu(0) and Cu_2_O, which, according to the literature, are positioned at 568.0 and 570.0 eV, respectively [39,40]. The major intensity peak recorded at 569.9 eV could be unequivocally assigned to Cu_2_O (Figure 5b).

Concluding this section, we can say that the CuNPs are in the form of needle-like aggregates, with a core principally composed of Cu(0) and a surface composed of Cu_2_O and CuO.

### 3.2. Incorporation of CuNPs in Porcine Ovarian Granulosa Cells Cultured with CuNPs

**Figure 6:**
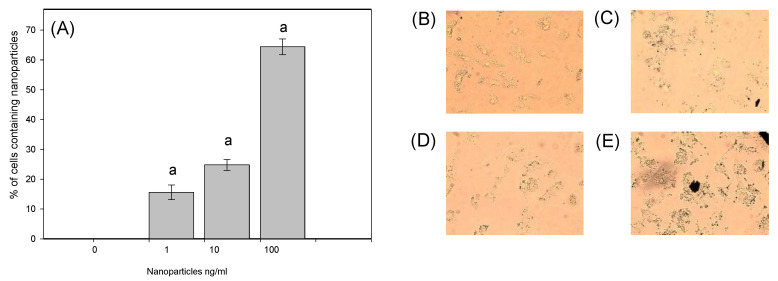
The incorporation of CuNPs into porcine ovarian granulosa cells cultured with CuNPs given at different doses. (**A**) The percentage of cells containing CuNPs. The results show (a) the effects of CuNPs: a significant (*p* < 0.05) difference between the cells treated and not treated with CuNPs (CuNPs at a dose of 0 ng/mL). The results are expressed as the mean ± SEM. (**B**–**E**) Representative images of cells cultured with CuNPs at different doses: 0 ng/mL (**B**), 1 ng/mL (**C**), 10 ng/mL (**D**), and 100 ng/mL (**E**).

After 48 h of co-culture of ovarian granulosa cells with CuNPs, some of the CuNPs were accumulated in the cytoplasm of the cells, some remained in the suspension, and some aggregated into CuNP clusters. This accumulation was increased by increasing the dose of CuNPs added (Figure 6).

### 3.3. CuNPs’ Effect on Ovarian Cell Functions

**Figure 7:**
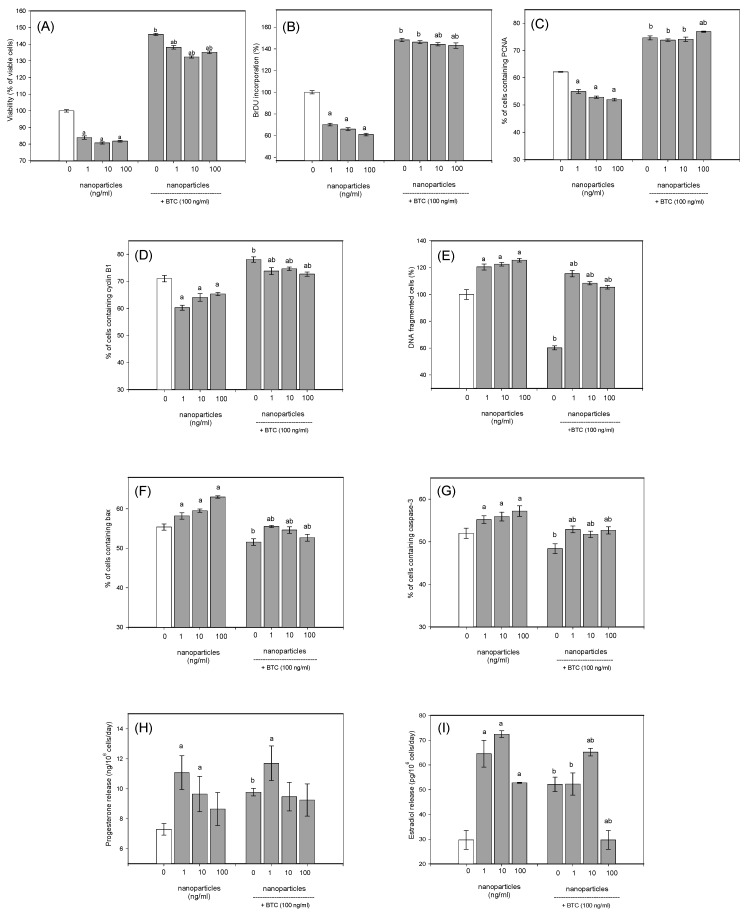
The effects of CuNPs, betacellulin (BTC), and both together on (**A**) cell viability, (**B**) BrdU incorporation, (**C**) PCNA accumulation, (**D**) cyclin B1, (**E**) fragmentation of DNA, (**F**) bax accumulation, (**G**) caspase 3 accumulation, and (**H**) progesterone and (**I**) estradiol release in cells from porcine ovary. The recorded data show (a) the effects of CuNPs, with a significant (*p* < 0.05) difference between the treated and untreated cells with CuNPs (CuNPs at a dose of 0 ng/mL); (b) the effect of BTC, with a significant difference (*p* < 0.05) between the cultured and non-cultured groups of cells with BTC. All the results are depicted as the mean ± SEM.

Upon adding CuNPs, a notable reduction in cell viability (Figure 7A), BrdU incorporation (Figure 7B), and the accumulation of both PCNA (Figure 7C) and cyclin B1 (Figure 7D) was observed, irrespective of the doses added. Conversely, CuNPs promoted the fragmentation of DNA (Figure 7E) and the accumulation of both bax (Figure 7F) and caspase 3 (Figure 7G) at all the doses added. CuNPs stimulated the secretion of progesterone (when given in doses of 10 or 100 ng/mL, Figure 7H) and of estradiol (at all the doses added, Figure 7I).

### 3.4. BTC’s Effect on Ovarian Cell Functions

BTC’s presence (see nanoparticles at 0 ng/mL + BTC group) notably enhanced cell viability (Figure 7A), the incorporation of BrdU (Figure 7B), and PCNA (Figure 7C) and cyclin B1 accumulation (Figure 7D). However, it decreased DNA fragmentation (Figure 7E), as well as bax (Figure 7F) and caspase 3 (Figure 7G) accumulation. Furthermore, BTC significantly stimulated the production of both progesterone (Figure 7H) and estradiol (Figure 7I).

### 3.5. BTC Prevents CuNPs’ Effect on Ovarian Cell Functions

The conducted experiments demonstrate the ability of BTC to modify CuNPs’ action. In the presence of BTC, CuNPs suppressed cell viability (at all the doses, Figure 7A), the incorporation of BrdU (at the doses of 10 or 100 ng/mL, Figure 7B), and the accumulation of cyclin B1 (at all the doses, Figure 7D), and stimulated DNA fragmentation (at all the doses, Figure 7E) and the accumulation of bax (at all the doses, Figure 7F) and caspase 3 (at all the doses, Figure 7G). In the presence of BTC, CuNPs did not affect the progesterone output (Figure 7H). CuNPs given at a dose of 10 ng/mL, together with BTC, promoted the release of estradiol, but reduced the estradiol output at a 100 ng/mL CuNP dose (Figure 7I). Therefore, the general patterns of CuNPs’ effects were retained in the presence of BTC.

On the other hand, cells cultured with CuNPs together with BTC showed higher cell viability (Figure 7A) and BrdU incorporation (Figure 7B), as well as higher accumulation of both PCNA (Figure 7C) and cyclin B1 (Figure 7D), than the cells treated only with CuNPs or the control cells. Moreover, in the presence of BTC, CuNPs inhibited BrdU incorporation only when given at 10 or 100 ng/mL, though not at all the doses (Figure 7B). In the cells cultured with CuNPs in combination with BTC, DNA fragmentation was lower than in the cells cultured only with CuNPs (Figure 7E). In the presence of BTC, a reduction in the accumulation of both bax (Figure 7F) and caspase 3 (Figure 7G) was noted below the CuNP-induced and even control levels. In the presence of BTC, CuNPs did not reduce PCNA accumulation, and, given at a 100 ng/mL dose, CuNPs even increased it (Figure 7C). Given together with BTC, CuNPs promoted progesterone release only at a 1 ng/mL dose (Figure 7H) and stimulated the estradiol output only at a 10 ng/mL dose (Figure 7I), but not at other doses. Therefore, the addition of BTC completely prevented the inhibitory effect of CuNPs on PCNA and the stimulatory effect of CuNPs on markers of apoptosis. Furthermore, it prevented the stimulatory effect of CuNPs on progesterone (at the doses of 10 and 100 ng/mL, Figure 7H) and estradiol (at a 100 ng/mL dose, Figure 7I) output. Finally, BTC not only prevented, but even reversed the inhibitory effect of CuNPs on PCNA (at 100 ng/mL, Figure 7C) and the stimulatory effect of CuNPs on estradiol (at 100 ng/mL, Figure 7I). Considering all these observations, it can be suggested that BTC can prevent and even invert the effect of CuNPs on ovarian cells.

### 3.6. CuNPs Prevent BTC’s Effect on Ovarian Cell Functions

The current study revealed the ability of CuNPs to modify the ovarian cell response to BTC. The stimulatory effect of BTC on cell viability (Figure 7A), BrDU incorporation (Figure 7B), the accumulation of PCNA (Figure 7C) and caspase (Figure 7G), and the secretion of progesterone (Figure 7H) and estradiol (Figure 7I) was found to be lower for cells cultured in the presence of CuNPs (at any dose) than for cells cultured without CuNPs. The same trend was observed for the inhibitory effect of BTC on the fragmentation of DNA (Figure 7E) and the accumulation of bax and caspase 3 (Figure 7F and Figure 7G, respectively). These results point to the ability of CuNPs to mitigate BTC’s effect on ovarian cells.

## 4. Discussion

### 4.1. CuNPs’ Effect on Ovarian Cell Functions

The outcome of CuNPs’ effect on the ovarian cells under study corroborates the inhibitory activity of these CuNPs on markers of cell viability and proliferation, and their stimulatory influence on apoptosis, reported previously [5,6]. However, the stimulatory effect of CuNPs on the release of estradiol and progesterone observed in the present experiments does not correspond with their inhibitory action observed previously. Progesterone and estradiol are considered to be the key markers of ovarian follicular health, atresia, and luteinization [29]. Therefore, the variation in the steroid hormone release observed in the present and previous experiments could be caused by differences in the initial state of the ovarian follicles and granulosa cells used in these experiments.

The capacity of CuNPs to down-regulate BrdU incorporation (a cell-proliferation marker) and the accumulation of PCNA (a promoter and marker of the mitosis S-phase) and cyclin B1 (a marker of M- and G-phases of mitosis) shown in the present study supports a suppressing role of CuNPs on the proliferation of ovary cells at the two cell-cycle phases. In contrast, the capacity of CuNPs to promote the fragmentation of DNA and accumulation of both caspase 3 and bax indicates its depleting effect on both intrinsic (cytoplasmic/mitochondrial) and extrinsic (nuclear DNA fragmentation) apoptosis. The down-regulation of proliferation and up-regulation of apoptosis by CuNPs could explain the ability of CuNPs to reduce ovarian cell proliferation, also observed in our previous experiments [5,6]. Finally, the CuNP-induced increase in progesterone and estradiol outputs suggest the possibility of CuNPs being a regulator of ovarian steroidogenesis and steroid-related events. In the present and past experiments, CuNPs were inhibitors of proliferation and viability, but promoters of ovarian cell apoptosis. Therefore, it is unlikely that these CuNPs’ effects could be mediated by progesterone and estradiol; these steroids are known to enhance ovarian cell proliferation while simultaneously inhibiting their apoptosis [29].

The capability of CuNPs to suppress the proliferation of porcine ovary cells, to enhance their apoptosis, and to alter their steroidogenesis, as deduced from the current and former assays, demonstrates the suppressive effects of these CuNPs on ovary cells, as well as the need to protect ovarian cells from their adverse effects. On the other hand, the lack of clear dose-dependence of CuNPs’ influence on some measured parameters, as well as the actual demonstration of CuNPs’ influence only on cultured porcine ovarian cells, indicate the necessity for further in vitro and in vivo studies of CuNPs’ effects on other models.

### 4.2. BTC’s Effect on Ovarian Cell Functions

The positive influence of BTC on PCNA, cyclin B1, and BrdU accumulation suggests that BTC enables enhancement of the proliferation of the cells from the porcine ovary by boosting some of the promoters of the two mitosis phases (G-phase and S-phase). These results are in agreement with earlier studies demonstrating BTC’s capacity to foster the proliferation of murine [14], chicken [18], and porcine [7] ovarian follicular cells. However, they contrast with the previously reported inhibitory effect of BTC on the proliferation of feline granulosa cells [20]. Furthermore, this demonstrates that BTC can inhibit both intrinsic and extrinsic apoptosis of the treated cells. This finding aligns with the earlier noted capability of BTC to diminish intrinsic apoptosis in feline ovary [20] and porcine ovary [7] cells. The positive effect of BTC on ovarian cell proliferation and viability, as well as on the release of estradiol and progesterone—which are known to promote ovary-cell proliferation and to inhibit their apoptosis—suggests that these steroid hormones might potentially facilitate the activity of BTEX in those processes. All in all, both current and previous findings highlight the straight and species-dependent impact of BTC on ovary cells. Furthermore, BTC’s ability to stimulate the proliferation, viability, and steroidogenesis of porcine granulosa cells, along with its inhibitory effect on extrinsic and intrinsic apoptosis, implies that BTC might directly enhance female reproductive processes in pigs. The current and previous [7] results can be considered proof of a direct stimulatory effect of BTC on porcine ovarian functions. To confirm its stimulatory effect and to better understand the mechanisms of BTC’s action, more in vitro experiments using BTC at various doses and analyses of more markers of ovarian cell functions are required. The next step, an assessment of the prospective applicability of BTC for boosting fertility in certain species, including pigs, requires specific in vivo experiments.

### 4.3. BTC’s Prevention of CuNPs’ Effect on Ovarian Cell Functions

The opposite behaviors of CuNPs and BTC on ovary-cell proliferation, viability, and apoptosis indicate their antagonistic character in the regulation of these processes. Furthermore, the current experiments showed BTC’s capability to prevent the effect of CuNPs on proliferation, viability, and apoptosis and, in some cases, on ovarian cell steroidogenesis. In some cases, BTC was able not only to prevent, but even to invert CuNPs’ effects. These observations support the fact that BTC cannot only stimulate ovary-cell functions, but also function as a natural protector against the toxic effect of these CuNPs. In our experiments, CuNPs were incorporated into cells cultured either with or without BTC. Therefore, it might be hypothesized that BTC does not affect the insertion of CuNPs into the cells, but rather mitigates, prevents, or inverts the effects of CuNPs within the cells.

Understanding the mechanisms of BTC’s influence on CuNPs’ effects is something that requires additional studies. Nevertheless, it might be proposed that BTC cannot chelate CuNPs in the medium because their uptake by the cells occurs either with or without the presence of BTC. The opposite effects of BTC and CuNPs on proliferation, viability, apoptosis, and steroidogenesis, as well as the ability of BTC to mitigate, prevent, and invert CuNPs’ effect on these processes, suggest that BTC can block CuNPs on intracellular regulators of proliferation, apoptosis, and steroid hormone release. Moreover, the ability of BTC, given at particular doses, to modify CuNPs’ effect on proliferation, viability, and apoptosis, but not on steroidogenesis, indicates that steroid hormones, despite their known influence on proliferation, viability, and apoptosis [29], are not the main mediators of CuNPs’ effects, and that CuNPs can influence the studied ovarian functions separately.

Taking into account the antagonistic behavior of BTC and CuNPs towards ovarian cells, the possible utility of BTC as a diagnostic marker of resistance of the reproductive system to environmental CuNPs, or as protector of this system against the suppressive influence of CuNPs, cannot be ruled out. This proposal requires additional validation through subsequent in vivo experiments on various animals by using different BTC preparations applied at different doses.

In summary, our initial findings suggest that CuNPs have a detrimental impact on the functions of porcine ovarian cells, while BTC seems to stimulate them. In addition, this outcome demonstrates that BTC can prevent and protect ovarian cells against the adverse effects of CuNPs. The potential applicability of these results in ecology, toxicology, reproductive medicine, and animal production is worthy of further examination.

### 4.4. CuNPs’ Prevention of BTC’s Effect on Ovarian Cell Functions

The results of the current experiments demonstrate the ability of CuNPs to reduce/mitigate the stimulatory effect of BTC on all the measured ovarian cell parameters. The present and previous observations demonstrate that BTC can be a relevant and potent physiological regulating agent of ovary-cell functions in various species [13,14,15,16,17,18,20]. The current investigation indicates that the environmental contaminants CuNPs can dysregulate reproductive processes not only via changes in the proliferation of ovarian cells, apoptosis, and steroidogenesis, but also through a reduction in the ovary-cells’ response to their physiological regulator BTC. Additionally, in cases where BTC could be applicable for preventing and treating ovarian cancer [19], CuNPs could reduce the efficiency of such BTC treatment. Should the influence of these CuNPs on BTC activity be validated under in vivo conditions, this effect must be taken into account during BTC application.

## 5. Conclusions

In conclusion, we have examined whether or not betacellulin (a stimulator of ovarian cell functions) can protect ovarian cells from the toxic (suppressive) influence of copper nanoparticles. The initial findings demonstrate the inhibitory effect of CuNPs and the stimulatory effect of BTC on cultured gilt ovary cells. This represents the first study evidencing the protective role of betacellulin against the reproductive toxicity of CuNPs; no similar in vivo or in vitro studies on any model have been reported yet. In addition, the data suggest that BTC and CuNPs can counteract each other’s effects. These effects and the mechanisms and nature of their functions, as well as their functional interconnections and their potential application, warrant additional in vitro and in vivo research across various species. Furthermore, the effect of long-term exposure to CuNPs requires elucidation. Unfortunately, it was not possible to investigate the long-term effects of nanoparticles and betacellulin on the primary ovarian cells used in the present experiments: these cells undergo luteinization and degeneration when cultured during several days. Nevertheless, the results of the present study could potentially be useful to understand, predict, control, and protect female reproductive functions against environmental contamination by CuNPs. Studies of this type are fundamental for the future development of diagnostic and therapeutic protocols in reproductive medicine.

## Data Availability

No new data were created or analyzed in this study. Data sharing is not applicable to this article.
